# Estimating the time of infection for African swine fever in pig farms in Korea

**DOI:** 10.3389/fvets.2023.1281152

**Published:** 2023-11-23

**Authors:** Hachung Yoon, Youngmin Son, Kyung-Sook Kim, Ilseob Lee, Yeon-Hee Kim, Eunesub Lee

**Affiliations:** Veterinary Epidemiology Division, Animal and Plant Quarantine Agency, Gimcheon, Republic of Korea

**Keywords:** African swine fever, pig farm, time of infection, simulation, Korea

## Abstract

African swine fever (ASF) is a highly contagious and lethal disease with characteristics of hemorrhagic fever. ASF outbreaks in pig farms significantly damage the entire pork industry. Understanding the transmission dynamics of ASF is crucial to effectively respond. Notably, it is important to know when the infection started on the outbreak farm. This study aimed at establishing a procedure for estimating the time of infection on pig farms affected by the ASF outbreak in Korea. The protocol for sampling to detect ASF virus infection, the estimation of the time interval between infection and detection, and the estimation of the infection stage parameters for the simulation model were described. After infection, fattening sheds (9.8 days in median) had the longest detection time compared with pregnant (8.6 days) or farrowing sheds (8.0 days). The intervals were 8.8 days for farrow-to-finisher farms, 7.0 days for farrow-to-weaning farms, and 9.5 days for fattening farms. The findings of this study provide valuable insights into ASF outbreaks in pig farms thus, improving the disease control ability.

## 1 Introduction

African swine fever (ASF) is a highly contagious and lethal disease affecting *Suidae* (domestic pigs and wild boars) and is characterized by hemorrhagic fever. It is caused by the ASF virus belonging to the *Asfarviridae* family (1). ASF is a disease listed by the World Organization for Animal Health, and its outbreaks in pig farms significantly damage the pork industry (2).

Since the 2018 ASF outbreak in China, it has spread to several Asian countries. In May and September 2019, an outbreak was reported in the Democratic People’s Republic of Korea (North Korea) and the Republic of Korea (South Korea, hereafter Korea), respectively. The first outbreak in Korea was reported in Paju, Gyeonggi-do Province, located approximately seven kilometers south of the border with North Korea (3–5). Fourteen outbreaks were confirmed in 2019, while only two outbreaks occurred in 2020. The number of outbreaks increased to five in 2021, followed by seven in 2022. As of July 2023, nine ASF outbreaks have been confirmed, bringing the total number of outbreaks to 37 since the index case in September 2019 (6). All outbreak farms were located in the northern part of the country (see ASF outbreak map at https://mafra.go.kr/FMD-AI2/map/ASF/ASF_map.jsp).

Upon ASF confirmation, all pigs on the outbreak farms were promptly culled and an outbreak investigation was conducted. Following the protocol of controlling ASF, vehicles, people, and goods entering and exiting the farm 21 days before the outbreak must be investigated, along with the usual livestock management and animal disease control on the farm (7). The outbreak investigation was conducted to identify the infection source, the introduction pathway of the virus to farms, and the farms at high risk in epidemiological relation to the current outbreak. The high-risk period, when the virus was most likely introduced into the outbreak farm and spread to other farms, is particularly a critical period requiring intensive investigation. To calculate this period, it is necessary to determine the time when the first infection occurred in animals on the outbreak farm. To estimate the likely time for an infection event, the evidence must be provided, and a basis for scientifically explaining the evidence is required (8). Moreover, the basis must be applied equally to all events. The criteria for estimating the infection time have already been established for the foot-and-mouth disease (9) and highly pathogenic avian influenza (10). No such study has yet been published on ASF. This study aimed at establishing a procedure for estimating the time of virus infection on pig farms affected by the ASF outbreak in Korea.

## 2 Materials and methods

### 2.1 Sample collection to detect African swine fever on pig farms

Detection of ASF outbreak farms in Korea is divided into two routes: reporting animals suspected of the disease and surveillance. Professionals in the livestock industry receive education repeatedly to promptly notify if any of the following applies: (1) death in sows and an increased number of stunted fattening pigs; (2) high fever over 40°C; (3) unexplained abortion or stillbirth; (4) daily mortality for all age groups higher than the average for the past 10 days (11). Sample collection following a report of suspected animals must include all dead and ill animals. Samples were blood from live animals and tissue from dead animals. Samples should also be collected from seemingly normal animals nearby (12). On the other hand, when conducting surveillance, blood sample is collected from 10 heads per farm at least once yearly from pig farms nationwide. In annual surveillance, samples are first collected from pigs in high-risk sheds and pens. The 10 heads comprise five sows and five fattening pigs. Additionally, when pigs are shipped out from farms located in intensive management areas, i.e., where the ASF virus was detected in wild boars, a test was also performed with blood samples. For shipping out fattening pigs, samples were collected from 10 heads. And all sows to be shipped out were tested (13).

Once a positive animal to ASF antigen test was identified, samples were additionally collected from the animals around it that looked normal. Blood samples were obtained from all sheds on the farm, 10 animals per shed, not only from those with positive animals (14). Samples were also obtained from additional dead animals found during the outbreak investigation, with oral and nasal swab sampling. Samples were tested using polymerase chain reaction (PCR) for antigen detection and enzyme linked immunosorbent assays for antibody detection. Details on the detection methods were described in the papers published (15, 16).

The proportion of antigen-positive animals, defined as prevalence, was calculated for each shed in which an antigen-positive animal was identified. The prevalence was calculated by including the pigs for which the presence of antigen was verified using PCR by the National reference Laboratory (Foreign Animal Disease Division of the Animal and Plant Quarantine Agency).

### 2.2 Estimating the time of infection

The time of the first infection of a herd in an ASF outbreak is estimated by considering factors, including mortality and antigen and antibody detection. Mortality is the first priority criterion and can be applied upon confirmation of the first ASF antigen-positive animal. When ASF infection was confirmed in a deceased animal, 1,000 random numbers following a Poisson distribution with the lambda parameter set as the time from infection to death were generated using the programing language R.^1^ The infection date for each animal with a confirmed infection was estimated by subtracting the number of days that corresponded to the quartile of the random numbers generated from the animal’s date of death.

For the second priority criterion, a program to simulate within-herd transmission was run to determine the date that predicts the number of antigen-positive animals, cumulative mortality, and the number of antibody-positive animals on the sampling day. By subtracting the number of predicted days from the sampling day, the time of the first infection in the shed was determined. If an antigen-positive animal was detected in more than one shed, the infection time was estimated per shed.

After estimating the infection time via mortality and simulation, the values were combined using random forest model written in R. However, the simulation program was run when there were at least three antigen-positive animals in a shed. For outbreak farms with antibody-positive animals, it was assumed that more than 10 days had passed since the infection onset, and the simulation results were compared for confirmation. However, the number of antibody-positive animals was not used to calculate the number of days after infection. Epidemiological factors, including people and vehicles entering and exiting the farm and events, were also considered in the final decision on the estimated infection time. Estimating the infection time on ASF outbreak farms was conducted for 34 of 37 confirmed ASF outbreak farms in Korea between 2019 and 2023. Two backyard farms and one farm with small number of native Korean pigs were excluded from the estimation.

### 2.3 Simulation program

The spread of the ASF virus within an infected pig shed was simulated using a mathematical modeling program with SLIR compartments: susceptible (S), latently infected (L), infectious (I), and removed (R). In this model, “removed” refers to the deceased or survived by developing antibodies. The simulation program was constructed using the programing language R. During the nn-day simulation period, the numbers of animals in the S, L, I, and R conditions and the number of newly infected animals were calculated for day i + 1. They were calculated based on the number of animals in each condition on the previous day (i), and the calculation was executed daily.

Latent period, number of days between infection and death, the number of days needed for antibody formation, and the percentage of dead or antibody-forming animals were entered as a constant in the model. Notably, a coefficient was needed to initiate simulation on the spread of infectious diseases. The rate at which new infections occur through contact between infectious and susceptible animals in a pig herd, was defined as “within-herd transmission coefficient.” The within-herd transmission coefficient was set to a unique value based on the herd type (0.9 for pregnant, 0.8 for farrowing, and 1.0 for fattening). The simulation was iterated, and the results were compared with real data (outbreak investigation and the animal study) to select the most appropriate values. Data from the outbreak investigation were records of daily mortality in the infected sheds. Regarding the animal study data, the time when the virus was detected in unvaccinated animals housed in the same pen as the inoculated animals was referenced (16). Estimation of other input values needed to run the program is discussed in Sections 2.4 and 2.5.

The simulation was executed with the number of animals to be simulated, assuming that the infection was started with one infectious animal then spread in the herd. The simulation program was iterated 1,000 times per execution.

### 2.4 Pathogenicity of the African swine fever virus

The parameter values for the infection stage of the ASF virus in pigs were estimated based on the results of the infection challenge experiments conducted using virus samples from ASF outbreak farms in Korea. The viruses in the pathogenicity experiments were 2019 Paju isolates (Korea/Pig/Paju1/2019), 2020 Hwacheon isolates (Korea/Pig/Hwacheon1/2020), 2021 Yeongwol isolates (Korea/Pig/Yeongwol/2021), 2021 Inje isolates (Korea/Pig/Inje1/2021), 2022 Hongcheon isolates (Korea/Pig/Hongcheon/2022), and 2023 January Pocheon isolates (Korea/Pig/Pocheon1/2023). The six isolates (i.e., the viruses isolated in the six outbreak farms) were injected intramuscularly into eight-week-old landrace pigs. As shown in Table 1, the study had six experimental groups with 22 animals (*n* = 3–5 per group). The experimental animals inoculated intramuscularly died within 10 days, and all animals in contact with them died within 18 days. Accordingly, the viruses were identified as highly virulent ASFVs that cause an acute clinical course and belonged to the p72 genotype II and CD2v serogroup 8 (15, 16).

**Table 1:** ASF outbreak status and experiments with infection challenge of ASF virus isolated from the outbreak farms.

Year	Number of ASF outbreaks confirmed in pig farms	Number of experiments with ASF virus from outbreak farms (Number of animals in each experiment)
2019	14	1 (3)
2020	2	1 (3)
2021	5	2 (3, 3)
2022	9	1 (5)
2023	7 (as of July)	1 (5)
Total	37	6 (22)

The experiments recorded the time (in days) until the onset of viremia (the presence of the virus in the bloodstream), detection of the virus in the oral or nasal cavity, and the onset of high fever with a body temperature of 40°C or over. The onset date of virus detection in the oral or nasal cavity was reported as “one to 2 days after the onset of viremia.” Therefore, for each animal, the onset date of virus detection in the oral or nasal cavity was estimated by adding a randomly assigned value of “one or 2 days” to the viremia onset date with R.

### 2.5 The infection stage parameters

The parameters for the infection stage required to run the simulation model were defined, and their values were calculated using the animal study results.

**Figure 1:**
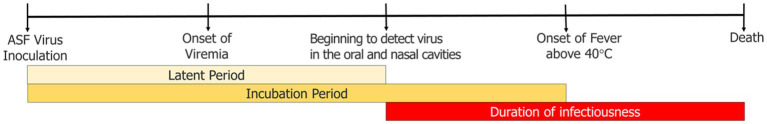
Definition of parameters for the ASF infection stage in pigs.

The ASF infection stages required to run the simulation model were defined as follows: (1) The latent period was defined as the interval from the day of inoculation of the virus into the experimental animals to the day the virus was first detected in the nasal or oral cavity; (2) The time from infection to death was defined as the interval between infection and death; (3) The duration of infectiousness was estimated from the beginning of virus detection in the nasal or oral cavity to death; (4) The incubation period was defined as the interval from the day of infection to the day when high fever with a body temperature of 40°C or more was measured (Figure 1).

Individual experimental measurements in each infection stage were combined as pooled means and 95% confidence intervals (95% CI). For this meta-analysis, “metaphor” package with mixed effect option was used in R. Both the characteristics of the six individual experiments (fixed effect) and overall variability (random effect) were considered through mixed effect model. The values of the infection stage were also expressed as probability density function. A goodness-of-fit test was conducted using “fitdistrplus package” in R to measure the difference between experimental measurements and several continuous probability distributions (i.e., Gamma, Logistic, Normal, Lognormal, and Weibull), the distribution with the smallest values of the Akaike information criterion and Bayesian information criterion was selected.

## 3 Results

### 3.1 Pig farms with outbreak of African swine fever

The 37 pig farms where ASF outbreaks were confirmed between 2019 and 2023 comprised 26 (70.3%) farrow-to-finisher farms, four (10.8%) farrow-to-weaning farms, and four (10.8%) fattening farms. The remaining three (8.1%) farms were one farm with Korean native black pigs and two backyard farms. Of the 34 commercial pig farms, the infected sheds where pigs tested positive for ASF virus antigens (referred to as antigen-positive animals) were found in 19 (55.9%) pregnant sheds, six (17.6%) farrowing sheds, and nine (26.5%) fattening (finisher) sheds. Outbreaks in sows (pregnant or farrowing pigs) accounted for 82.4% (28 farms) of the total. The number of pigs in the infected sheds, antigen-positive animals, and deaths increased in the pregnant, farrow, and fattening sheds order. Conversely, based on the number of pigs per shed, the highest prevalence was observed in pregnant sheds. Table 2 shows the data on the infected sheds in ASF outbreak farms in Korea. Antibodies were detected in one farm with positive cases in farrowing sheds and three farms with positive cases in fattening sheds. No cases of antibody detection were reported in pregnant sheds. The two farms with positive cases in the fattening sheds were excluded from antibody tests.

**Table 2:** Status of ASF-infected sheds on pig farms confirmed between 2019 and 2023 in Korea.

Infected Shed	Number of outbreak farms	Number of pigs in infected shed (A)^*^	Antigen-positive pigs		Number of deaths in relation to ASF
			Number (B)^*^	Prevalence (B/A, %)^*^	
Pregnant	19	107 (91, 223)	6 (4, 11)	3.9 (2.2, 6.9)	2 (0, 3)
Farrow	6	126 (78, 262)	8 (5, 10)	2.3 (2.0, 11.4)	3 (1, 8)
Fattening (Finisher)	9	332 (227, 665)	10 (3, 11)	1.2 (0.8, 5.0)	4 (0, 6)
Total	34^1^	180 (101, 351)	6 (4, 11)	2.8 (1.5, 6.7)	2 (0, 5)

1 Exclude two backyard farms and one farm with native Korean black pigs.

* Quartile statistics: median (1st quartile, 3rd quartile).

### 3.2 Values for the infection stage of African swine fever

Table 3 shows the values for the infection stages of ASF. For the latent period, the range was 2–5 days, with a pooled mean (95% CI) of 4.3 (3.7–4.8) days. For the incubation period, a mean of 4.3 (3.4–5.2) days was estimated, and the range was 3–7 days. For the infection time to death, the range was 4–10 days, and the mean was 9.0 (8.9–9.1) days. Consequently, the duration of infectiousness ranged from 1 to 6 days, with a mean of 4.4 (3.6–5.2) days.

**Table 3:** Infection stage duration parameters estimated based on the experiment of ASFV infection challenge.

Infection stage parameter	Values^*^	Pooled mean (95% CI)	Probability distribution
Latent period	4 (4, 5)	4.3 (3.7–4.8)	Lognormal (meanlog = 1.5, sdlog = 0.2)
Incubation period	5 (4, 5)	4.3 (3.4–5.2)	Normal (mean = 4.7, sd = 1.1)
Time to death	9 (8, 9)	9.0 (8.9–9.1)	Lognormal (meanlog = 2.1, sdlog = 0.2)
Duration of infectiousness	5 (5, 6)	4.4 (3.6–5.2)	Weibull (shape = 3.7, scale = 4.5)

* Estimated based on experimental observation, Quartile statistics: median (1st quartile, 3rd quartile).

### 3.3 Simulation of within-herd transmission

The simulation program was executed using the first quartile (Q1), median, and third quartile (Q3) values of the number of pigs in infected sheds in Table 2 and the coefficient of transmission based on shed type. The simulation output revealed that the spread of ASF virus infection was relatively faster in small herds compared to large herds. Although the absolute number of infected individuals differed based on herd size and shed type, the trends of increase, peak, and decrease at each stage of infection were similar. The period with the highest daily number of newly infected animals was 16–18 days for Q1, 17–19 days for the median, and 19–22 days for Q3 values of the number of pigs. The highest prevalence was below 40% in sows (38.3–39.0% for pregnant sheds and 35.7–36.6% for farrowing sheds) and above 40% (40.7–41.4%) for fattening sheds. The highest prevalence reached at 23–24 days for Q1, 24–26 days for the median, and 26–28 days for Q3 values of the number of pigs in the shed.

The ASF-confirmed deceased pigs were regarded as being infected 7–11 days before death, which was the interquartile range of the random values generated (with lambda = 9), the median value of time to death (Table 3).

**Figure 2:**
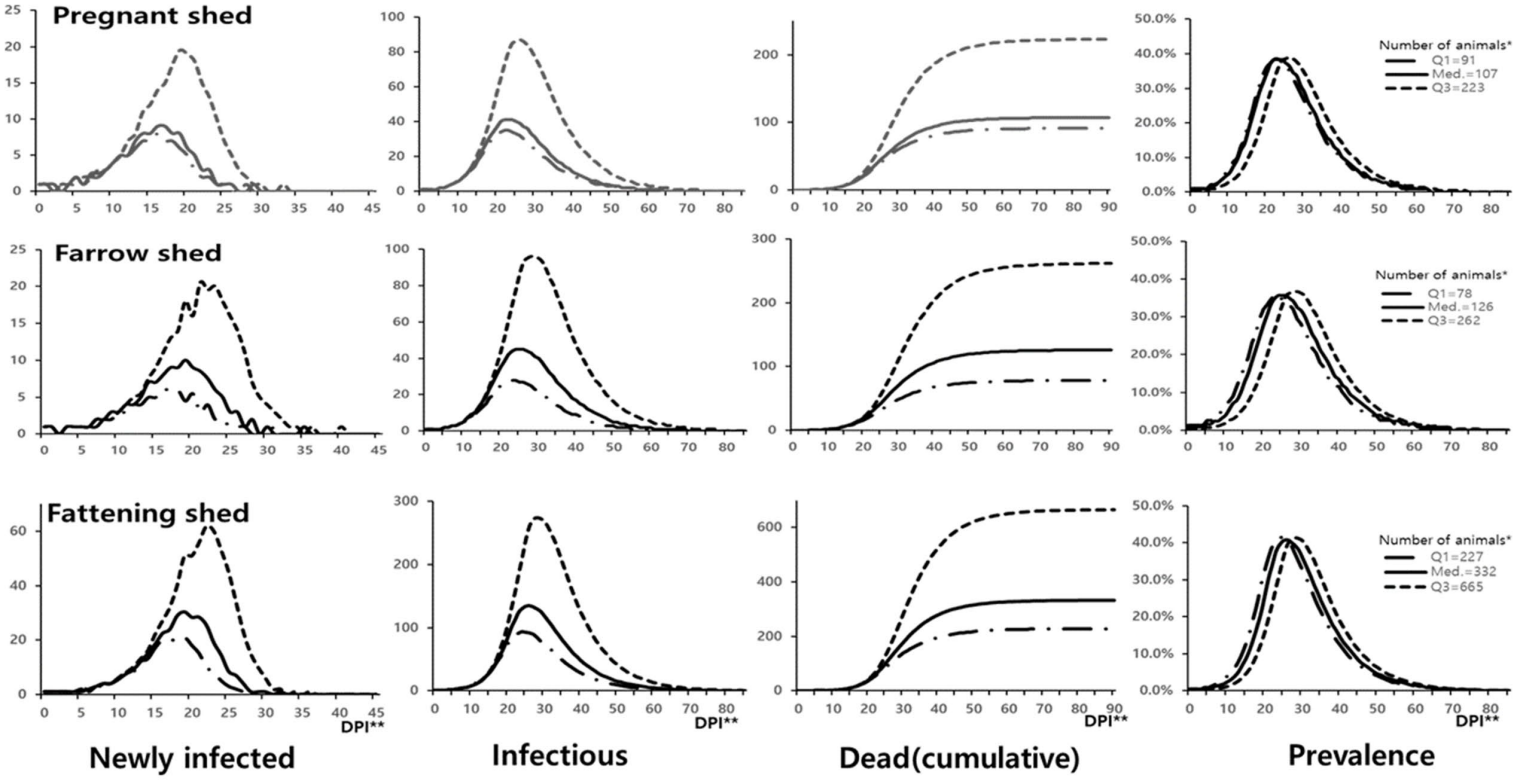
Within-herd transmission of ASF infection according to the type of infected shed. *Number of animals Q1: 1st quartile, Med.: Median, Q3: 3rd quartile. **DPI: Days post introduction (start of infection).

Following the infection in the animals and spread within the pig herds, it was expected to reach the prevalence of detection (shown in Table 2) at 8–11 days in pregnant sheds (3.9%), 5–11 days in farrowing sheds (2.3%), and 6–10 days in fattening sheds (1.2%). Supposing the ASF outbreaks were not recognized and no response measures were implemented, then, the entire herd was expected to die within 64 days (for the median number of pigs) in pregnant sheds, 70 days in farrowing sheds, and 77 days in fattening sheds from the time of infection (Figure 2).

### 3.4 Time of infection on the African swine fever outbreak farm

The time interval from infection to detection was estimated to be a median of 9.0 (Q1–Q3, 7.8–10.5) days in ASF outbreak farms in Korea. The median (Q1–Q3) intervals were 8.6 (7.8–10.5) days in pregnant sheds, 8.0 (7.9–8.6) days in farrowing sheds, and 9.8 (9.0–12.8) days in fattening (finisher) sheds. In real outbreak, the fattening sheds had the longest detection time after infection. When considering the livestock type on the outbreak farms, the median (Q1–Q3) intervals were 8.8 (7.8–12.8) days for farrow-to-finisher farms, 7.0 (3.9–10.1) days for farrow-to-weaning farms, and 9.5 (7.9–11.3) days for fattening farms (Table 4).

**Table 4:** Time from infection to detection in the ASF outbreak pig farms in Korea between 2019 and 2023.

Infected pigs		Number of outbreak farms	Time (days) from infection to detection^*^
Shed	Pregnant	19	8.6 (7.8, 10.5)
	Farrow	6	8.0 (7.8, 8.6)
	Fattening (Finisher)	9	9.0 (9.8, 12.8)
Farm	Farrow-to-finisher	26	8.8 (7.8–12.8)
	Farrow-to-weaning	4	7.0 (3.9–10.1)
	Fattening	4	9.5 (7.9–11.3)
Total		34^1^	9.0 (7.8, 10.5)

1 Exclude two backyard farms and one farm with native Korean black pigs.*Quartile statistics: median (1st quartile, 3rd quartile).

## 4 Discussion

An understanding of within-herd transmission dynamics is important for an effective response to an infectious animal disease and requires knowledge of when the infection started on the farm. Simulation models can be particularly helpful in such cases (17, 18). If the herd size is small and the number of cases is low, then, the infection time can be estimated based on the number of days since the onset of the oldest appearing clinical symptoms or lesions, and the incubation period. However, if many animals such as pigs or poultry are housed together, and many have become ill or died simultaneously, simulation can be used to predict the number of newly infected, infectious, and dead animals on daily basis. The values of the input parameters affect the prediction accuracy (19–21).

Based on the infection challenge using the ASF virus isolated from farms in Korea between 2019 and 2021, it was found that all viruses belonged to the strain causing an acute form of illness (15). The ASF virus from outbreaks in 2022 and 2023 was also determined to be in the acute form. Viremia was detected 2–5 days after inoculation, followed by detection in the nasal or oral cavity within 1–2 days, and death occurred 4–9 days later (15). Similar experiments conducted in other countries showed that viremia was detected 2–5 days after infection challenge, followed by detection in oral, nasal, or rectal swabs within 1–2 days. The incubation period until clinical symptoms, such as high fever, appeared at 3–5 days and death occurred at 6–10 days after inoculation (22–25). The results of our study, which calculated a 95% CI, were consistent with that of studies with infection challenges in Korea and other countries. The estimated time until virus detection in the oral or nasal cavity ranged from 3.7 to 4.8 days, the incubation period ranged from 3.4 to 5.2 days, and mortality ranged from 8.9 to 9.1 days (Table 3).

Viremia was detected 10–13 days after inoculation in pigs that were in contact with virus-inoculated pigs (22, 24). Pigs in contact with the inoculum developed clinical symptoms after 9 days (25) and 6 to 7 days after inoculation (26). The duration of infectiousness was 3.6–5.2 days in our study compared with 2.9 days from the nasal cavity and 3.2 days from the oral cavity with the virus from Georgia 2007 (22). The infectious period ranged from 2 to 9 days in Europe (27).

Studies on within-herd transmission between pigs have mainly focused on direct contact (28, 29). However, indirect transmission through viruses in the environment can still occur. For instance, an experiment conducted in Poland showed that healthy pigs that entered a pen emptied for 1 day after being occupied by ASF-infected pigs, exhibited severe clinical symptoms within a week (30). In pig farms in Korea, pigs have contact with each other within the same pen, but there is more indirect contact with pigs in other pens through human behavior, including tool usage. The reproductive number (R_0_), representing transmission between pigs, has been reported to be 2.8 (95% CI 1.3–4.8) within pens and 1.4 (0.6–2.4) between pens (31). The R_0_ for within-herd transmission varies from 1.6 to 24.2 in different studies, based on the breeding type and measurement method (32). In our study, both direct and indirect transmission routes were considered when determining the within-herd transmission coefficient. The smallest coefficient was assigned to the farrowing shed, where the farrowing sow stays in an individual stall with her suckling piglets, with a value of 0.8. Based on the Enforcement Rule of the Livestock Industry Act, In Korea, pregnant sheds are required to be in the form of grouping pens by 2029 (33). As of 2023, the transition of the pregnant pig shed from stall to grouping pen has commenced. Consequently, the transmission coefficient for the pregnant shed was set to 0.9. The within-herd transmission coefficient was the highest for fattening pigs (1.0), where pigs could come into close contact with pen mates.

Based on this study, it is estimated that ASF can be detected after 8.0 (95% CI 7.8–8.6) days from infection in farrowing sheds, 8.6 (7.8–10.5) days for pregnant sheds, and 9.0 (9.8–12.8) days for finishers at farrowing sheds (Table 4). Various factors, such as the coefficient used, the number of animals, and the work patterns in the pig farms may have influenced the simulation results. In Korean ASF outbreak farms, the estimated infection-to-detection time (7.8–10.5 days) was found to be shorter than the period suggested by a Danish study (13–19 days) (18) and an experiment using a moderately virulent virus (more than 20 days) (28). The relatively rapid detection in Korea may be attributed to the intensive breeding system and careful identification of ill and dead pigs. Detecting and reporting animals showing abnormalities, such as sudden death, is crucial for early ASF detection and containment. A study that reconstructed the spread patterns within a large-scale pig farm in Latvia based on an ASF investigation suggested that the first infected animal died within a week after infection, but went unnoticed (34). In Korea, sudden death is the most frequently observed symptom reported by farmers in ASF outbreak farms (35). The Korean government has established criteria for reporting suspected ASF cases, which include sudden death in sows and daily mortality higher than the average for the last 10 days in all age groups (11). Prompt reporting of a deceased animal observed on a pig farm effectively enhances the efficiency of ASF response.

Besides ASF-related deaths, this study calculated the prevalence of antigen-positive populations compared with the total number of pigs in the infected sheds. Although laboratory tests were not conducted on all pigs in the affected sheds, specimens were obtained from all ill and deceased pigs and their cohabitants, ensuring that the most visibly affected animals were included in the tests. It took 9.0 (range 7.8–10.5) d for 2.8% (95% CI 1.5–6.7%) of the pigs in the sheds to test positive for the ASF antigen (Tables 2, 4).

## 5 Conclusion

In this study, a systemic procedure for estimating the time of introduction of ASF virus into a pig farm upon the confirmation of an ASF outbreak on the farm was established. The procedure was also applied to estimate the time of infection and the time interval from infection to detection (i.e., the period during which there is a risk of unknowingly releasing the virus from the outbreak farm). The findings provide valuable insights into ASF outbreaks in pig farms, particularly those with intensive management systems, such as those in Korea. This study will help facilitate early ASF detection and implementation of preventive measures, thus improving the ability to control and manage the disease.

## Data availability statement

The raw data supporting the conclusions of this article will be made available by the authors, without undue reservation.

## Author contributions

HY: Conceptualization, Data curation, Formal analysis, Funding acquisition, Investigation, Methodology, Project administration, Resources, Software, Visualization, Writing – original draft, Writing – review & editing. YS: Formal analysis, Software, Writing – review & editing. K-SK: Data curation, Formal analysis, Software, Writing – review & editing. IL: Formal analysis, Writing – review & editing. Y-HK: Resources. EL: Funding acquisition, Supervision, Writing – review & editing.

## Footnotes

^1^ https://www.r-project/org
